# Illustrated Catalogue and Phylogenetic Relationships of 330 Species of Arctiinae Moth Species from the Chocó Rainforest in NW Ecuador: Most Species are Undescribed

**DOI:** 10.1007/s13744-025-01333-y

**Published:** 2025-12-22

**Authors:** Gunnar Brehm, Dennis Böttger, Ugo Mendez Diniz, David A. Donoso, Mareike Kortmann, Jörg Müller, Dominik Rabl, Alexander Keller, Michel Laguerre

**Affiliations:** 1 Institute for Zoology and Evolutionary Biology, Phyletisches Museum, Friedrich-Schiller-University, Jena, Germany; 2 Plant-Insect Interactions, School of Life Sciences, Technical University of Munich, Freising, Germany; 3 Departamento de Biología, Escuela Politécnica Nacional, Quito, Ecuador; 4 Grupo de Investigación en Ecología y Evolución en los Trópicos -EETrop, Universidad de Las Américas, Quito, Ecuador; 5 Department of Animal Ecology and Tropical Biology, Biocenter, University of Würzburg, Rauhenebrach, Germany; 6 Environmental Agency Austria, Spittelauer Lände 5, 1090 Vienna, Austria; 7 Cellular and Organismic Networks, Faculty of Biology, Ludwig-Maximilians University Munich, Planegg-Martinsried, Germany; 8 Léognan, France

**Keywords:** Lepidoptera, Tropical rain forest, Insect diversity, Identification

## Abstract

Tropical rain forests are the most species rich terrestrial habitats on Earth, but their insect diversity is understudied, and it is unclear how many species are already scientifically described. A model group to study description patterns are tiger moths (Erebidae: Arctiinae), a species-rich moth clade that comprises subtaxa that differ considerably in appearance. We inventoried Arctiinae moths in a lowland rainforest in the Canandé and Tesoro Escondido Reserves, NW Ecuador, and sorted 12,335 individuals into 330 species, of which 303 had DNA barcode (COI) data extracted. We found 52 species of Lithosiini, 4 species of Arctiina, 17 species of Pericopina, 132 species of Phaegopterina, 52 species of Euchromiina and 71 species of Ctenuchina. A total of 45% of the species can be assigned by us to known named species, but the numbers vary considerably within the subtaxa: while in the conspicuous butterfly-like Pericopina 82% are described, this figure is only 26% for the smaller and cryptic Lithosiini, indicating a strong description bias even within a relatively well-known group of macromoths. This may indicate that particularly small and inconspicuous moth species have so far been neglected and that museum collections might often not be representative archives of insect diversity. Therefore, more systematic and non-biased collection campaigns should be carried out for better estimates of insect diversity. All 330 Arctiinae species are listed in three electronic catalogues, which contain all barcoded individuals as well as corresponding type material from museums, allowing a transparent and straightforward verification of all identifications. We constructed a preliminary phylogeny using literature data as backbone in combination with our DNA COI sequence data which provides a unique and useful data base for future studies in the Chocó rainforest.

**Supplementary Information:**

The online version contains supplementary material available at 10.1007/s13744-025-01333-y.

## Introduction

Tropical rain forests are the most species rich terrestrial habitats on earth, and insect diversity is highest in the tropics (Erwin 1982; García-Robledo et al. 2020). These, as well as most other terrestrial habitats on Earth, cannot function properly without insects due to their key roles in foodwebs as predators, parasitoids, prey, herbivores, detrivores and pollinators (Cock et al. 2013). At first glance, a simple question is how many species there are in these habitats and how many of them are already known (or unknown) to science. However, at a global scale, estimates of global biodiversity still vary considerably (Ødegaard 2000). Due to the increasing loss of intact habitats (e.g. primary forests), accelerated by the effects of climate change, many species will likely disappear from the planet even before they can be counted and described: it is a race against time (Lopez-Vaamonde et al. 2019).

Despite these difficulties, case studies on insect diversity at the local or regional scale can still provide important indications of the numbers of species and proportions of undescribed species that can be expected in tropical rainforests. For instance, Brehm et al. (2011) estimated that around 90% of the species in the tropical and small sized genus *Eois* (Lepidoptera: Geometridae) are still undescribed, which means that in addition to the approximately 230 known *Eois* species, there are probably another 2000 undescribed species. This percentage is much lower in more conspicuous and better-known Lepidopteran taxa such as butterflies, Saturniidae and Sphingidae of which it is assumed that most species are already known (Kitching et al. 2018). While it is hardly feasible to conduct a broad comparison of description patterns in all Lepidoptera (let alone all insects), one group could serve as an example, in which both relatively large and colourful species are represented (which look very similar to butterflies), as well as many small and inconspicuous species. One such group are tiger and lichen moths (Erebidae: Arctiinae), as this taxon comprises subtaxa that differ substantially in appearance. While many are relatively colourful and conspicuous/aposematic, there are also many cryptically coloured species (Fiedler & Brehm 2021). Arctiinae are a large group of moths; there are around 11,000 described species known worldwide, including more than 6000 species in the Neotropics (Watson & Goodger 1986). Arctiinae diversity has been studied in different Neotropical regions (e.g. Teston & Corseuil (2004), Hilt et al. 2006, Brehm 2007, 2008, Teston et al. (2012), Moreno & Ferro (2016) Jaimes Nino et al. 2019, Böttger et al. 2025, Zenker et al. (2015) and they are assumed to be important pollinators in tropical rainforests (Diniz et al. 2025).

### Towards Reliable Species Catalogues

In order to answer the question of how many species there are and how many of them are known to science—even for a manageable group like Arctiinae—requires some effort. Only counting operational taxonomic units (OTUs), e.g. from DNA metabarcoding, is relatively simple, because it is not necessary to link libraries to reliable taxonomic information. Reliable linking with valid taxonomic data is complex and time consuming, but opens the avenue for deeper analyes, with focus on traits (e.g. Jaimes Nino et al. 2019). Moreover, many tropical insect species have not yet been recorded in databases such as BOLDSystems (e.g.Brehm et al. 2016; Lopez-Vaamonde et al. 2019; Ratnasingham et al. 2024), and even if they are, the existing identifications often appear contradictory and unreliable. Well-curated insect sequence libraries appear to be still an exception rather than the rule. For example, even in the well-known European Lepidoptera fauna, a large proportion of cryptic diversity was found in southern European Gelechiidae moths (Huemer et al. 2020). Especially for tropical insects there is usually hardly any summarized and illustrated literature, and many references are old and outdated. The study of type specimens is therefore often the only way to achieve a reliable comparison between newly collected moths and existing taxa (Brehm et al., 2011). Sometimes, types have been illustrated (e.g. Pinheiro 2016), but it is still necessary to visit museums and take photos. It has proven to be a good practical method for tropical macromoths to create an overview using electronic catalogues. For instance, such catalogues have been published for Arctiinae and Geometridae moths from a Peruvian lowland rainforest (Jaimes Nino et al. 2019), for Colombian Geometridae (Murillo-Ramos et al. 2021), and for Neotropical Geometridae genera (Brehm et al. 2019).

### The Need for Phylogenetic Data

To understand the evolution of organisms, information about their phylogenetic relationship is essential. For example, if traits of moths (such as size and lightness) are being studied, it is necessary to know the relationship between the insects to correctly interpret the results (Westoby et al. 2023). Here, too, tropical insect communities face greater challenges. Unlike well-known vertebrates such as birds, the phylogenetic relationships between insects at species level are generally unknown. Due to the short length of the COI sequences (658 base pairs), we do not expect them to provide enough information to construct a phylogeny that is correct in every detail. However, in Neotropical Arctiinae, there is at least a phylogeny available that includes most Neotropical genera (Zenker et al. 2016). This makes it possible to construct the backbone of a phylogeny onto which COI data can be mapped (Kortmann et al. 2025).

In this paper, we sought to investigate how many species of Arctiinae are found in a biodiversity hotspot, i.e. in two reserves in tropical lowland rainforest of the Chocó-Darien ecoregion (NW Ecuador), and how many of these species can be assigned to described species. Furthermore, we assessed whether there is evidence for a relationship between the probability that a species is described and its conspicuousness and size. The main product of this paper is to provide the first catalogue of the currently known Arctiinae of the Chocó region, in which all species are properly illustrated – together with available images of type material. Finally, we also aim to provide a provisional phylogeny, specifically for the Arctiinae of the Chocó forest, which will be an important basis for further ecological analyses of insect diversity in the region.

## Material and Methods

### Field Collection

Moths were collected between 2022 and 2023 in the Río Canandé Reserve (0.523746° N, 79.210391° W) and the Tesoro Escondido Reserve (0.541917° N, 79.144972° W), Esmeraldas province, NW Ecuador in lowland forest ecosystems in the Chocó-Darien ecoregion (Fagua & Ramsey 2019), with an annual temperature of 21–24 °C and annual rainfall of ca. 4000–5000 mm (Escobar et al. 2025). Most moths were quantitatively collected along a chronosequence of 62 plots (0.25 ha) ranging from active disturbance agricultural sites to regenerating secondary forests and old-growth forests (Escobar et al. 2025). In sites of old-growth forests (*N* = 17) and late secondary forests (older than 20 years, *N* = 12) where vertical stratification was present, both in the understorey and canopy of the forest were sampled (Böttger et al. 2025; Diniz et al. 2025). Furthermore, all reassembly plots (understorey) were sampled in November 2021 or 2022 (Müller et al. 2023). Arctiinae were sampled together with Saturniidae, Sphingidae, Geometridae and Hedylidae in the area, and represented most of all collected individuals. Light trapping methods with portable UV lamps and funnel traps are described in detail by Brehm (2017), Singh et al. (2022), Böttger et al. (2025) and Diniz et al. (2025).

### Processing and Identification

Moths were carefully spread and photographed using near-daylight LEDs and a 10-mm scale bar; a detailed description of photography methods is provided by Brehm (2025). All individuals were numbered and databased using a code scheme ‘EcEs-Lep-nnnnn’ where nnnnn represents a unique identifier. One leg per specimens of all species were sampled stored in 96% ethanol with subsequent sequencing performed at the Canadian Centre for Genomics (Guelph, Canada), with successful extraction and amplification for 92% of all species. Moths were identified using reference collections from Costa Rica, Ecuador and Peru and photographs of material taken at museums by GB and ML, with the most important collections being the Natural History Museum (London, UK) and the National Museum of Natural History (Washington D.C, USA). In addition, we also used literature, particularly illustrated catalogues (e.g. Pinheiro & Gaal-Haszler (2015) and Silva et al. (2023)). It must be emphasized that all identifications are hypotheses. Since the taxonomy of Neotropical Arctiinae obviously requires a high number of generic revisions, and we have found a significant number of previously undescribed species (see Results), all identifications are subject to uncertainty. Taxonomic names were checked using published taxonomic literature, supported by the website http://ftp.funet.fi/index/Tree_of_life/ where almost all species names are linked to the original descriptions (and if available to original illustrations).

We categorized identifications into five levels: (1) species were identified at species level if there was an extremely high similarity in external morphology between the collected specimen and the type specimen. The type locality was considered an important information, for example making a species match more likely if the respective type specimen was collected in the same region—i.e. in (western) Ecuador and Colombia. For instance, there are several similar species (some probably undescribed) around *Gorgonidia buckleyi* Druce. Since our specimen looks like the type specimen, and this specimen was collected in Ecuador, we assume that it is conspecific. (2) Species were identified at species group level if there was an extremely high similarity between the collected specimen and the compared type specimen, but the species is also part of a complex of very similar species. For example, we found a species closely resembling *Eucereon tarona* (Hampson), but we found similar looking species (from other regions)—and found it impossible to decide which is conspecific with the type specimen. (3) We assigned the category ‘near species’ in all cases when a species looks similar as a described species but also shows distinct features. In many cases, the type locality did not match well either. Taxonomically, this is the same as genus-level identification, but from a practical viewpoint, it is usually more helpful to assign a (presumably) closely related species as a reference. In some cases, it was possible to assign a species near to two different species and chose one of them. (4) If no resembling species was found in collections or the literature, we assigned most remaining species to genus level. We are aware that genus-level identification can be erroneous, especially given the problem that many Neotropical Arctiinae genera are not well defined and/or monophyletic (see Discussion).

### Catalogues

The catalogues were produced by GB in Adobe InDesign 2024 using photographs taken from collected specimens by GB and from type material in museums by GB and ML. Catalogue pages have the format A4 (297 × 210 mm), and each regular page shows one species. We did not find obvious mismatches between our sorting to morphological criteria and BINs (Barcode Index numbers). With a few exceptions, each species in our dataset is represented by a different BIN. A catalogue page comprises (when available) data on genus, species, species number, author, year, status, depository of the type specimen(s), the BIN, countries in which conspecific specimens were recorded (i.e. same BIN, checked in BOLD), remarks and number of sequenced individuals from the study area. On each species page, photos of all DNA-barcoded individuals with their respective individual numbers, a photo of a type specimen of the respective species (if not available, another museum specimen) that allows direct comparison, and optionally further reference specimens are provided.

### Phylogeny

Phylogenies were constructed at the level of tribes and subtribes using the study of Zenker et al. (2016) as backbone. We also provide an unconstrained phylogeny for comparison. It is known that Pericopina and Phaegopterina are not monophyletic (e.g. Jacobson and Weller 2002; Zenker et al. 2016; Dowdy et al. 2020; Moraes et al. 2021), and we therefore assigned all species to one of the clades within these taxa (named ‘partim 1’, ‘partim 2’, …). For other analyses, we have nevertheless combined the respective branches of both taxa for practical reasons. COI sequences were aligned within each of the above mentioned clades, using the AlignSeqs function from the DECIPHER package (Wright 2015). We estimated phylogenetic trees with the TreeLine function using maximum likelihood with the GTR+G4 model. The subtrees were then added to the backbone tree at the respective node with the bind.tree function from the ape package (Paradis & Schliep 2019). Approximate Bayesian (aBayes) branch support probabilities for each clade are reported in supplementary material x. To calibrate all branch length after the grafting process, we used the compute.brlen function from ape. Construction of the phylogenetic tree followed the protocol described in Kortmann et al. (2025).

## Results

### Proportion of Described Species

Using morphological criteria and results from DNA barcoding, we sorted the available material of 12,335 moth individuals to 330 Arctiinae morphospecies, which will be henceforth treated as species (described or not described). Three hundred three (92%) of the species were successfully DNA barcoded. The morphological sorting matched the COI sorting (using BINs from BOLDSystems) in almost all cases. In nine cases, we defined species with two BINs because no morphological differences were recognizable, although a thorough analysis including genitalia dissection could yield further insights. However, this is beyond the scope of our paper. We are not aware of cases in our dataset where one BIN represents more than one species but cannot exclude that such cases were overlooked.

**Fig. 1:**
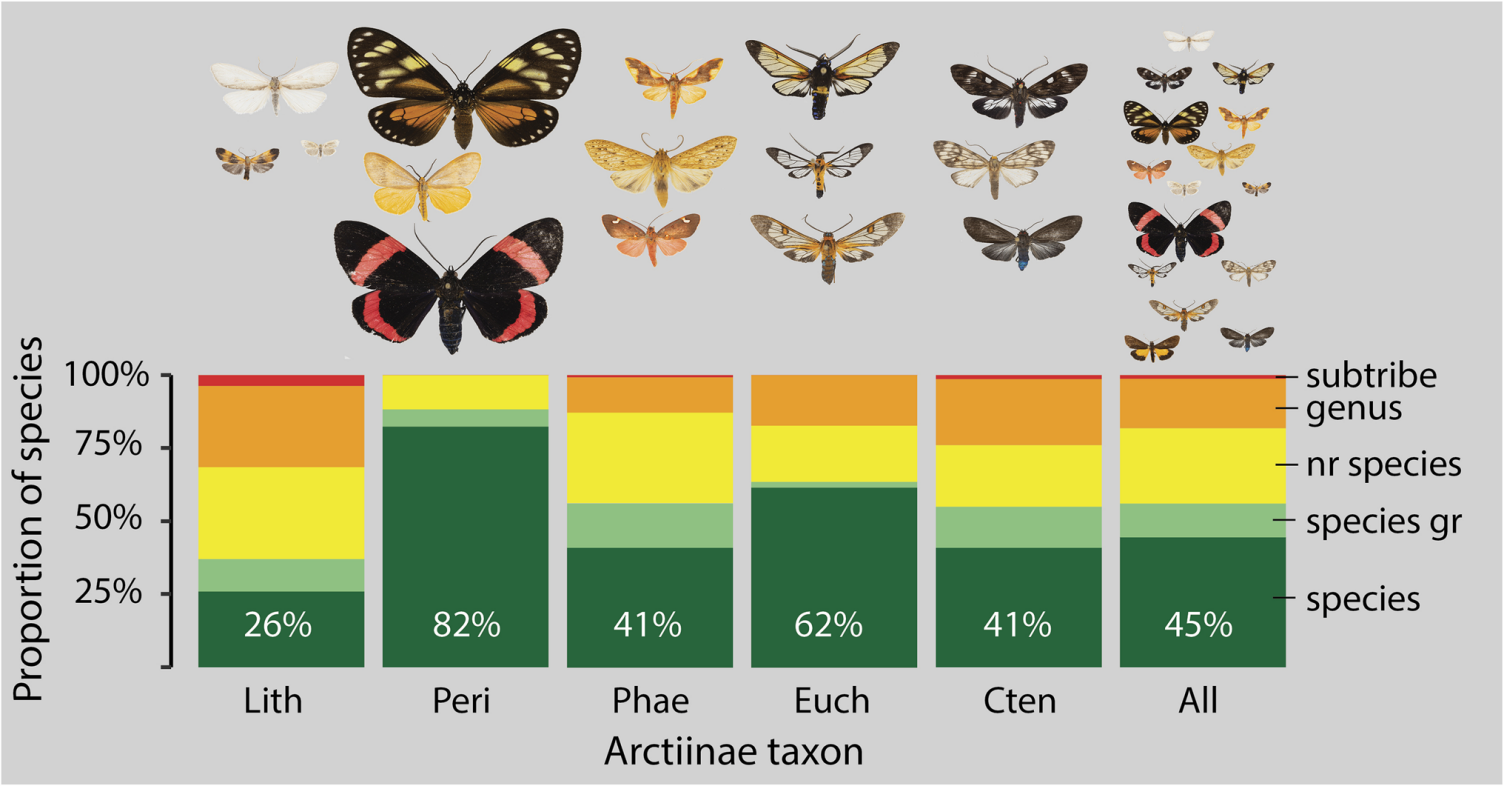
The proportion of described species in Arctiinae sampled in Chocó rainforest, NW Ecuador, differ considerably between the taxonomic subgroups. The percentages of species level identifications are shown for each of the subtaxa and the entire Arctiinae: Lith, Lithosiinae; Peri, Pericopina; Phae, Phaegopterina; Euch, Euchromiina; Cten, Ctenuchina. nr species, near species; species gr, species group. The subtribe Arctiina is only represented by four species and was therefore omitted. The highest percentage of species-level identification is found in Pericopina—a group that is characterized by butterfly-like large and conspicuous species, whereas the lowest percentage is found in Lithosiini which is considered a taxonomically difficult group with numerous similar species

For all Arctiinae, we identified 99% at least at the genus level; only four species could not be associated with a genus. It must be noted, however, that some identifications at genus level must be regarded as provisional since many genera appear not well defined and/or non-monophyletic (see Discussion). Forty-four percent were identified at the species level, 12% at the species group level, 26% were identified near a described species and 17% at the genus level (Fig. 1). There were considerable differences between the subtaxa. For instance, species-level identification was smallest in lichen moths (Lithosiini) with only 26% identified at the species level and highest in Pericopina in which 82% were assigned to a named species.

### Catalogues

**Fig. 2:**
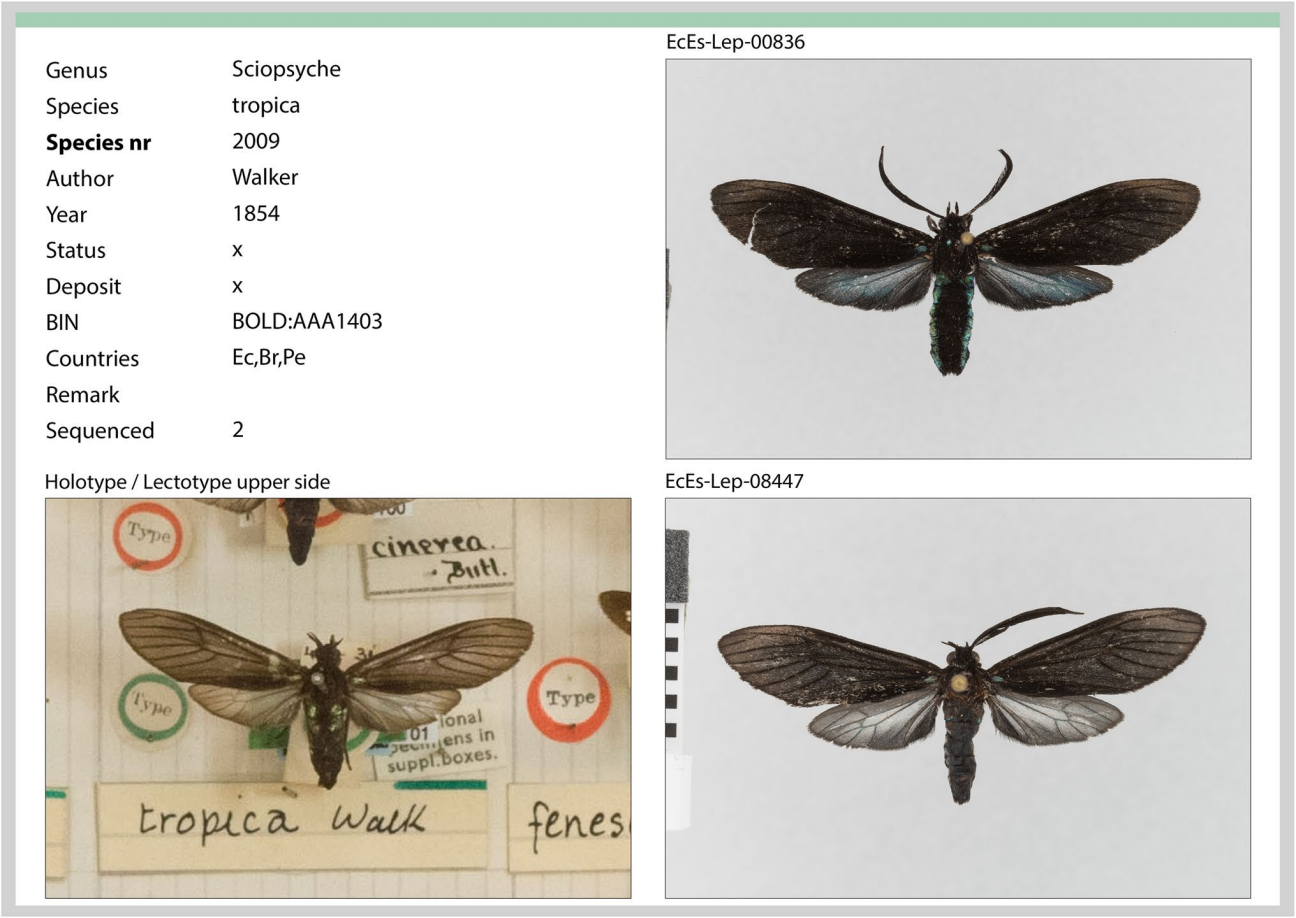
Example of catalogue page (upper half shown) of *Sciopsyche tropica* (Walker) with available taxonomic information (genus, species, author, year of description), deposit of type specimen (NHM, Natural History Museum London, UK), status (usually empty), BIN (barcode index number), countries in which the species is known to occur (i.e. records with the same BIN in Boldsystems), here Ecuador, Brazil and Peru; and the number of sequenced species from the study area. The DNA-barcoded specimens are shown (on the right, with their unique specimen IDs) as well as a corresponding photo of a type specimen (on the left, here, the holotype)

Three illustrated catalogues with all Arctiine species occurring in the area are provided in the supplementary material. Catalogue 1 contains Lithosiini, Arctiina and Pericopina; catalogue 2 contains Phaegopterina and catalogue 3 contains Ctenuchina and Euchromiina (links provided in the supplementary material, see below). An example page is shown in Fig. 2 with available taxonomic information, deposit of type specimen, BIN, countries in which the species is known to occur (i.e. records with the same BIN in Boldsystems, non-exhaustive), and the number of sequenced species. The DNA-barcoded specimens are shown (with their unique specimen IDs) as well as a corresponding photo of a type specimen (in the example, the holotype of *Sciopsyche tropica* (Walker)).

### Species Richness and Phylogeny

**Fig. 3:**
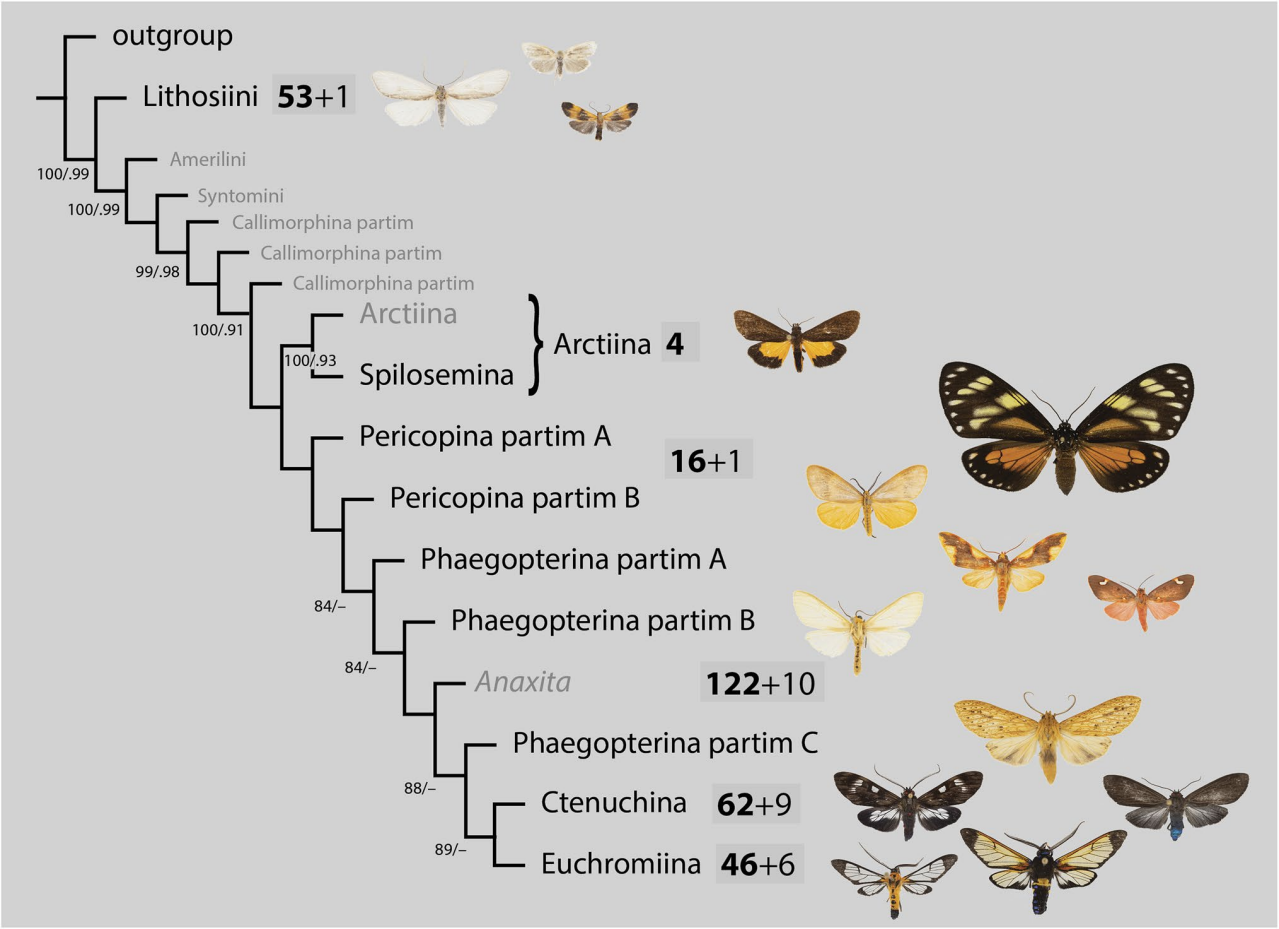
Phylogeny of the major Arctiinae clades, extracted from from Zenker et al. (2016). Numbers next to the branches are bootstrap values and posterior probabilities (only bootstrap values > 80 shown). Black font: taxa present in the study area, grey font: taxa not known in the study area. Pericopina and Phaegopterina probably represent paraphyletic groups. Spilosomina is considered here a synonym of Arctiina. Bold face: Number of DNA barcoded species in the study area, regular font + number species not barcoded. Sum of all species recorded in the study area: 330

A total of 330 species were assigned to one of the larger subtribes within Arctiinae: 54 to Lithosiinae, 4 to Arctiina, 17 to Pericopina, 132 to Phaegopterina, 71 to Ctenuchina and 52 to Euchromiina. The backbone phylogeny derived from Zenker et al. (2016) is depicted together with example images in Fig. 3. The phylogeny including 303 DNA-barcoded species is provided in the supplementary material.

**Fig. 4:**
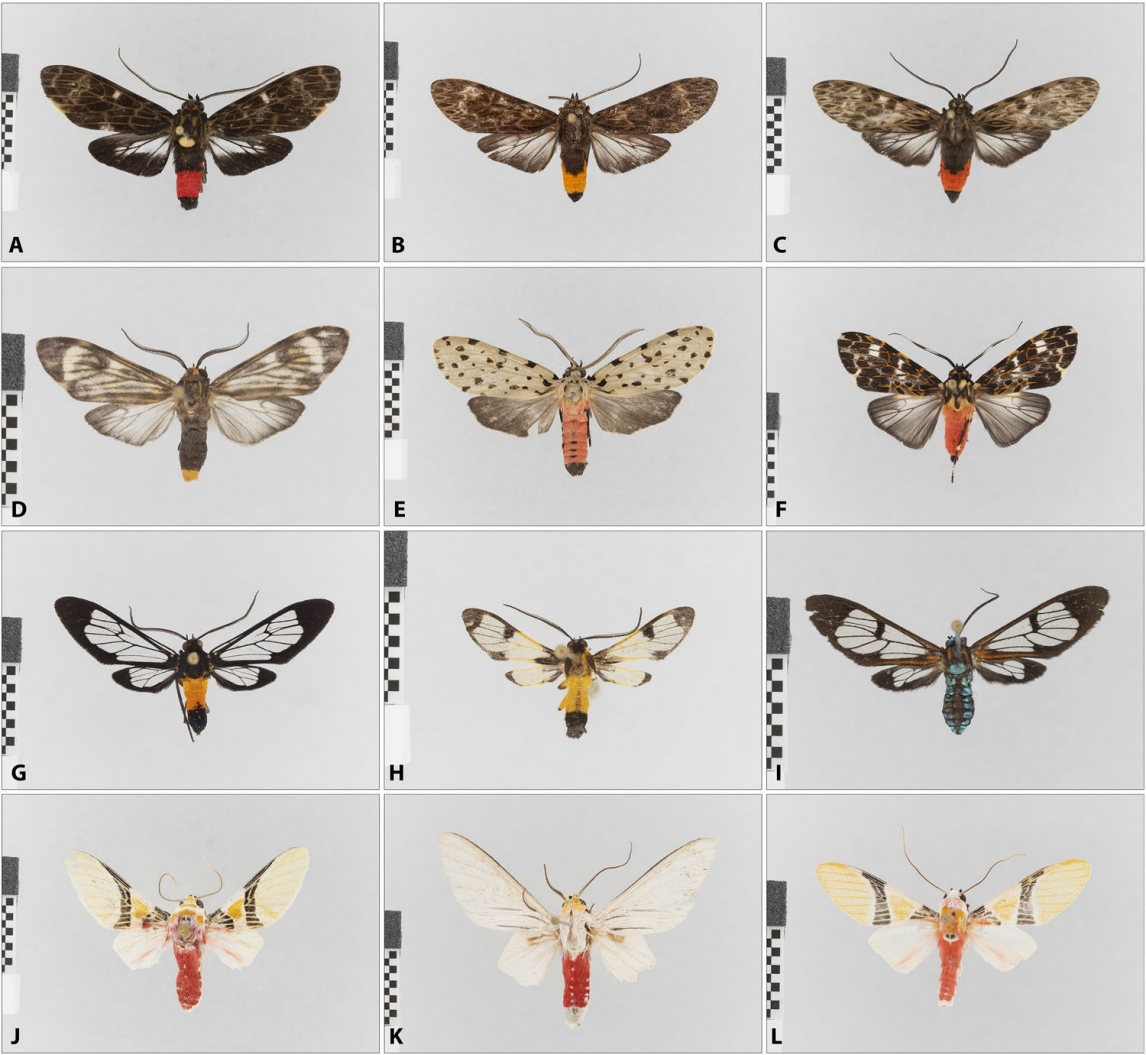
Examples of Arctiinae that apparently require taxonomic revision, derived from the results of the phylogeny (Fig. 2 and Fig. S1). **A**–**C** Apparently closely related species currently placed in three different genera. **D**–**F** Apparently not closely related species currently placed in *Eucereon.* **G**–**J** Apparently not closely related species currently placed in *Cosmosoma.* **I**–**K** Apparently closely related species currently placed in *Idalus* and *Eupseudosoma.* **A** *Acridopsis* near *varia* (Walker). **B** *Stollius amadis* (Schaus). **C** *Laguerreius* near *pseudarchias* (Schaus). **D** *Eucereon aroa* Schaus. **E** *Eucereon dognini* Rothschild. **F** *Eucereon* near *tarona* Hampson. **G** *Cosmosoma stilbosticta* (Butler). **H** *Cosmosoma semifulva* (Druce). **I** *Cosmosoma remota* (Walker). **J** *Idalus iragorri* group (Dognin). **K** *Eupseudosoma* near *involuta* (Sepp). **L** *Idalus* near *carinosa* (Schaus). Scale bar 10 mm, white reflectance standard 95%, dark grey reflectance standard 10%

In the phylogeny (supplementary material), the taxa are predominantly arranged in such a way that closely related species probably also have short distances in the tree. For example, all twelve *Agylla* Walker species are grouped together, as are most of the other genera, respectively. A closer look at the phylogeny reveals that there is at least one case in which—judging by the great external morphological similarity—apparently closely related species are placed in three different genera (Fig. 4A–C). In more cases, however, genera appear in different places in the phylogeny. This is the case, for example, with *Talara* Walker, *Prepiella* Schaus (both Lithosiini), *Idalus* Walker, *Scaptius* Walker, *Amaxia* Walker (all Phaegopterina), *Eucereon* Hübner (Ctenuchina) (Fig. 4D–F) and *Cosmosoma* Hübner (Euchromiina) (Fig. 4G–I). In other cases, certain genera are nested in other groups, for instance *Macroptila* near *monstralis* Schaus in an otherwise homogeneous clade of twelve *Agylla* species, and *Atyphopsis* near *modesta* Butler nested in *Correbidia* Hampson. In the unconstrained phylogeny (supplementary material), all taxa (tribes, subtribes) are split into several subunits without exception; the extreme case is represented by the Pericopina, which are divided into two larger clades and six species widely scattered in the tree.

## Discussion

Our study represents the first systematic survey of Arctiinae in the Chocó rainforest. With 330 species locally, the group is very species rich. The number is similar to that reported by Jaimes Nino et al. (2019) for a lowland rainforest in Peru (332 species), but the analyzed number of individuals in Peru was considerably lower and did not include canopy samples. In our study, Arctiinae were recorded in studies both in the canopy and in the understory at more than 60 sites in several field seasons (Böttger et al. 2025; Diniz et al. 2025). Other authors found fewer Arctiinae species in their study areas, for example Teston et al. (2012) found 78 species in an Amazonian rainforest fragment, Teston & Corseuil (2004) found 192 species in southern Brazil, Moreno & Ferro (2016) found 149 in the Cerrado biome and Zenker et al. (2015) found 294 species in a study area in the Atlantic rain forest. In all of these studies, including ours, (many) other species have probably not yet been discovered, as is usual in tropical insect communities. It must also be considered that a certain number of Arctiinae species are largely diurnal and cannot, or only in small numbers, be recorded by light trapping, and that, e.g. bait attraction (and other methods) could contribute additional species (Boppré & Monzon 2023).

### Proportion of Described Species

Our results suggest more than half of the Arctiinae species from the study area are still undescribed. This is not entirely surprising for tropical insect communities. In microlepidoptera, these values can be much higher; for instance, 499 out of 507 BINs collected in Malaise traps in Madagascar were novel to BOLD, indicating a very low proportion of described species (Lopez-Vaamonde et al. 2019). The example of Arctiinae shows that the proportion of described species depends on how conspicuous the insects are. While in the butterfly-like Pericopina 82% are described, this figure is only 26% for the smaller and much less conspicuous Lithosiini, which is likely to indicate strong description bias even within a relatively well-known group of macromoths. The proportion in the other groups range from 41 to 62%, indicating that the Hymenoptera-mimicking Euchromiina have been somewhat more popular for taxonomists than Ctenuchina and Phaegopterina. It is also conceivable that the patterns found are partly due to the fact that the more inconspicuous groups exhibit a high degree of morphological similarity among themselves (such as *Agylla*), and that only the recent analysis of DNA sequences has provided a realistic picture of diversity. Our results correspond to personal observations that can be made in almost all museum collections known to the authors, i.e. large and colourful insects are usually disproportionately overrepresented. Another observation is that taxonomists do not describe new species at random, but according to their personal preferences, and that these preferences are often (but not always) orientated towards the attractiveness of the insects. A bias in favour of aesthetically attrative species was also found, for example with regard to the conservation status of European butterflies (van Tongeren et al. 2023). It therefore seems to correspond to natural human behaviour that ‘more beautiful’ species receive attention first, even if many of the smaller Lithosiinae species reveal colours and patterns that are very attractive to humans at second glance. For most collections, however, this means that small and inconspicuous species have so far been neglected and that museum collections are not a representative archive of insect diversity: small and inconspicuous species are not only scientifically undescribed, but they may not even been collected yet in many cases. We encourage further quantitative studies on these questions. For example, new automated methods open the possibility of quantifying the size and colourfulness of Lepidoptera on a mass scale (Correa Carmona et al. 2025), thus allowing statistically sound results to be obtained that we cannot provide with our study. And of course, we also encourage museums to collect more representatively than before and to systematically collect the small and inconspicuous species as long as this is still possible (Lopez-Vaamonde et al. 2019).

### Catalogues

If poorly studied tropical taxa are included in ecological or phylogenetic studies without any further documentation than a molecular sequence, there is a risk that results can be misleading because taxa were not correctly identified. Such errors cannot easily be found and may persist for long periods (Brehm et al. 2019). Illustrated species catalogues can overcome these problems by making species identifications fully transparent and therefore verifiable than the publication of pure species lists (e.g. Zenker et al. 2016; Jaimes Nino et al. 2019; Brehm et al. 2019, Murillo-Ramos et al. 2021).

All identifications in the catalogues must be regarded as provisional because in most cases, a fully reliable identification could only be achieved in an in-depth analysis of the taxon, including the study of many individuals per species, the examination of all relevant literature and the investigation of the genitalia, apart from fundamental questions about taxonomic concepts. However, this was far beyond the scope of our study. Alternatively, identification could be achieved through DNA-barcoded-type specimens, but these are so far only available for recently described species (e.g. Brehm 2018). DNA barcodes (or other genomic information) are not available for most older insect type specimens although exceptions exist such as the geometrid *Eois* (Strutzenberger et al. 2012). The major challenge of species identification represented by one or a few individuals is the discrimination between interspecific and intraspecific variability. While colour is often relatively variable within species, patterns are usually rather species-specific (GB, ML, own observations). Sexual dimorphism in Arctiinae is usually moderate but strong dimorphism occurs, for example in Pericopina (Moraes et al. 2021) and in *Agylla* lichen moths (Weller et al. 1999). COI barcoding is particularly useful here, but identification can be strongly impeded if, for example, a male (with sequence data) is compared with a female type specimen (without sequence data). We understand some of our identifications will eventually proof to be wrong, or that identifications at genus level or near species level can be improved in future studies. However, there is a tradeoff between higher quality standards (including dissection, taxonomic revision, etc.) and the possibility to publish catalogues in a reasonable time. The catalogues make it possible for the first time to access one of the region’s major lepidopteran groups by researchers, the local population and visitors in western Ecuador and Colombia. We intend to update the catalogues in the future through further studies in the region over the next few years in the Reassembly research project.

### Phylogeny

The phylogeny shows predominantly meaningful and plausible results. However, we would like to emphasize that this is only a working basis that allows us to analyze local species communities in a meaningful way—and not a comprehensive, universally applicable phylogeny for Arctiinae. This assessment is possible because all species were also morphologically assessed and identified, regardless of the sorting based on sequence data. However, it is not to be expected that the relatively short COI sequence data will produce exclusively meaningful results. There are some outliers whose actual phylogenetic position deviates significantly from expectations. Moreover, although 330 species are included, taxon sampling at the generic level is far from complete which can lead to incorrect groupings. However, the phylogeny points to some existing problems with current taxonomy, as illustrated in Fig. 4. In many cases, generic names are found in different places in the tree. In some cases, this may be a methodological artefact. For example, two apparently closely related *Idalus* species are located at different positions in the tree (Fig. 4K, M). On the other hand, the tree also shows that certain generic assignments should be reconsidered. For example, it is not clear how the existence of the genera *Idalus* and *Eupseudosoma* can be justified—at first glance, synonymisation seems reasonable. The genera *Acridopsis*, *Stollius* and *Laguerreius* (Fig. 4A–C) group closely in the phylogeny and are externally similar. However, their genitalia structures are largely divergent which was the reason of their recent creation (Cerda 2020). We leave open the question of whether this split has more advantages than disadvantages; in any case, the clade around *Eucereon* should be further taxonomically investigated, as as exemplified, for example, by Pinheiro (2016). Another example is *Cosmosoma*, in which, despite taxonomic works in recent years (Laguerre 2014a, 2014b), taxa are still grouped together that probably do not form natural entities (Fig. 4G–I). These examples demonstrate that neither the backbone-COI phylogeny always provides correct results, nor does the existing taxonomy represent a system of natural groups in all cases. Nevertheless, the availability of a phylogeny generally represents a major advance that allows to assess how and why communities of species differ from random expectations for evolutionary and ecological relatedness (Emerson & Gillespie 2008). The comparison between the backbone-constrained tree and the unconstrained tree demonstrates that the integration of a better-resolved phylogeny is obviously important and an unconstrained COI tree is likely to cause misinterpretations. We would very much welcome the publication of further phylogenetic studies on Neotropical (and global) Arctiinae that examine the phylogenetic relationships even more robustly than before and cover as many genera as possible.

## Supplementary Information

Below is the link to the electronic supplementary material.

Supplementary file 1 (DOCX 15.1 KB)

## Data Availability

All relevant data are published as supplementary material along with this paper.
