# Supplementary material for: Illustrated Catalogue and Phylogenetic Relationships of 330 Species of Arctiinae Moth Species from the Chocó Rainforest in NW Ecuador: Most Species are Undescribed
Source: Neotrop Entomol. 2025 Dec 22;54(1):127. doi: 10.1007/s13744-025-01333-y (PMC12722485; doi:10.1007/s13744-025-01333-y)
Supplement: Supplementary file 1 — (DOCX 15.1 KB) [file 13744_2025_1333_MOESM1_ESM.docx]

**Illustrated catalogue and** **phylogenetic relationships of 330 species of Arctiinae moth species from the Chocó rainforest in NW Ecuador: most species are undescribed**

Gunnar Brehm^1*^, Dennis Böttger^1^, Ugo Mendez Diniz^2^, David A. Donoso^3,4^, Mareike Kortmann^5^, Jörg Müller^5^, Dominik Rabl^5,6^, Alexander Keller^7^ and Michel Laguerre^8^

^1^ Institute for Zoology and Evolutionary Biology, Phyletisches Museum, Friedrich-Schiller-University Jena, Germany

^2^ Plant-Insect Interactions, School of Life Sciences, Technical University of Munich, Freising, Germany

^3^ Departamento de Biología, Escuela Politécnica Nacional, Quito, Ecuador

^4^ Grupo de Investigación en Ecología y Evolución en los Trópicos -EETrop-, Universidad de las Américas, Quito, Ecuador

^5^ Department of Animal Ecology and Tropical Biology, Biocenter, University of Würzburg, Rauhenebrach, Germany

^6^ Environmental Agency Austria, Spittelauer Lände 5, Vienna 1090, Austria

^7^ Cellular and Organismic Networks, Faculty of Biology, Ludwig-Maximilians University Munich, Planegg-Martinsried, Germany

^8^ 31 rue de la Haute-Lande, 33850 Léognan, France

*Corresponding author, [gunnar.brehm@uni-jena.de](mailto:gunnar.brehm@uni-jena.de)

**Supplementary Material**

Links:

<https://github.com/DesBoe/Illustrated-catalogue-and-preliminary-phylogeny-of-330-species-of-Arctiinae-moth-species>

Catalogues 1–3 (pdf)
Maximum likelyhood tree with 303 species (pdf) with backbone
Maximum likelyhood tree with 303 species (pdf) without backbone
Species list (Excel file)

Data on BOLD will be released with publication.

Additional material: Approximate Bayesian (aBayes) branch support probabilities for each Arctiinae clade
